# Anti-tumour effects of all-trans retinoid acid on serous ovarian cancer

**DOI:** 10.1186/s13046-018-1017-7

**Published:** 2019-01-08

**Authors:** Noor A. Lokman, Rachel Ho, Kavyadharshini Gunasegaran, Wendy M. Bonner, Martin K. Oehler, Carmela Ricciardelli

**Affiliations:** 1 Discipline of Obstetrics and Gynaecology, Adelaide Medical School, Robinson Research Institute, University of Adelaide, Adelaide, South Australia 5000 Australia; 2 Department of Gynaecological Oncology, Royal Adelaide Hospital, Adelaide, South Australia 5005 Australia

**Keywords:** Serous ovarian cancer, Annexin A2, S100A10, All- trans retinoic acid

## Abstract

**Background:**

Annexin A2 is increased in serous ovarian cancer and plays an essential role in ovarian cancer invasion and metastasis. In combination with S100A10, annexin A2 plays an important role in the plasminogen activator system regulating plasmin production. The aim of this study was to investigate the potential utility of all-trans retinoid acid (ATRA), an inhibitor of the annexin A2-S100A10 signalling pathway, as a new therapeutic against serous ovarian cancer.

**Methods:**

In this study we determined the effects of ATRA treatment (1-5 μM) on annexin A2 and S100A10 expression, plasmin activation, and the ability of ATRA to inhibit serous ovarian cancer cell survival, motility and invasion in vitro. We also employed an ex vivo tissue explant assay to assess response to ATRA treatment in serous ovarian cancers. Cryopreserved serous ovarian cancer tissues were cultured on gelatin sponges for 72 h with ATRA (1 μM). Effects on apoptosis and proliferation were assessed by immunohistochemistry using antibodies to cleaved caspase 3 or Ki67, respectively.

**Results:**

Survival of serous ovarian cancer cells (OVCAR-3, OV-90, & OAW28) was significantly decreased by ATRA treatment (1-5 μM). ATRA (1 μM) also significantly decreased proliferation (Ki67 positivity, *p* = 0.0034), S100A10 protein levels (*p* = 0.0273), and increased cell apoptosis (cleaved caspase-3 positivity, *p* = 0.0024) in serous ovarian cancer tissues using the ex vivo tissue explant assay. In OAW28 cells, reduced cell survival following ATRA treatment was associated with a reduction of S100A10 mRNA and protein levels, S100A10 and annexin A2 membrane localization, plasmin generation, motility and invasion. In contrast, ATRA inhibited OV-90 cell survival and invasion but did not affect plasmin activation or S100A10 and annexin A2 expression or membrane localization.

**Conclusions:**

These findings suggest that ATRA inhibits serous ovarian cancer proliferation and invasion via both S100A10 dependant and S100A10 independent mechanisms. Our results show that ATRA has promising potential as a novel therapy against serous ovarian cancer that warrants further evaluation.

**Electronic supplementary material:**

The online version of this article (10.1186/s13046-018-1017-7) contains supplementary material, which is available to authorized users.

## Background

Ovarian cancer is ranked the fifth leading causes of cancer death in women and is also the most lethal gynaecological malignancy in the developed world [1, 2]. Despite improvements in surgery and systemic treatments, including a broad range of new chemotherapies and targeted therapies, the survival rate has not considerably changed over the last 20 years [2]. Up to 75% of women are diagnosed at advanced stage (stage III and IV) and it is projected that there will be approximately 22,440 new cases and 14,080 deaths due to ovarian cancer in 2017 in the United States [2]. Currently, the 5 year survival rate after diagnosis is only 29% at advanced stage, with 52% of the deaths occurring in women aged 60 years and above [2]. More effective molecularly targeted therapies to improve survival are therefore urgently required.

Ovarian cancer has the predisposition to metastasize by invasion into mesothelium that lines the organs within the peritoneal cavity and mediate local invasion and metastasis to abdominal organs and structures such as the bowel and omentum [3–5]. To identify novel therapeutic targets involved in ovarian cancer metastasis, our laboratory investigated the interaction of ovarian cancer and peritoneal cells using proteomics [6, 7]. Annexin A2 was identified by mass spectrometry to be up-regulated by ovarian cancer-peritoneal cell interactions [8, 9].

Annexin A2, a multi-functional phospholipid calcium-binding protein is involved in the plasminogen activation pathway [7, 8]. Annexin A2 exists both as a monomer and a heterotetramer known as AIIt [7, 10]. The annexin A2 monomer is an intracellular 38 kDa protein, whilst the AIIt heterotetramer complex consisting of two subunits of annexin A2 monomers and two subunits of S100A10 (also known as p11) is localized on the plasma membrane [7, 11]. There is emerging evidence showing an important role of the AIIt heterotetramer in the plasminogen activator system [7, 12–14]. The interaction between annexin A2, S100A10 and tissue plasminogen activator (t-PA) mediates the conversion of plasminogen to plasmin which facilitates the extracellular matrix (ECM) degradation, matrix metalloproteinase (MMP) activation, epithelial to mesenchymal transition (EMT) and angiogenesis, leading to increased cancer cell migration and invasion [8, 15, 16].

Our previous studies reported that annexin A2 plays an important role in ovarian cancer invasion and metastasis [8] and increased expression of both annexin A2 and S100A10 is associated with poor serous ovarian cancer outcome [9]. The aim of this study was to investigate an inhibitor of the annexin A2 signalling pathway, all-trans retinoid acid (ATRA), for its efficacy to inhibit serous ovarian cancer cell survival and invasion.

ATRA is an active vitamin A metabolite which is currently used as a primary treatment for patients with acute promyelocytic leukemia (APL) [17]. APL is a distinct subtype of acute myeloid leukemia that expresses the promyelocytic leukemia-retinoic acid receptor alpha (PML-RAR-alpha) oncoprotein and is characterized by severe bleeding resulting from increased plasmin production [17–20]. ATRA has been shown to be beneficial in inducing differentiation and promoting apoptosis of leukemic cells and improving bleeding symptoms by inhibiting plasmin production and decreasing expression of annexin A2 and S100A10 [21–23]. Due to its relatively low systemic toxicity there is a great interest in expanding the therapeutic use of ATRA in other cancer types [24–26].

While the effects of ATRA on ovarian cancer proliferation and apoptosis have been previously investigated [27–31], to date no data has been reported on the effects of ATRA on both annexin A2 and S100A10 expression in serous ovarian cancer cells. This study evaluated the effects of ATRA on annexin A2 and S100A10 expression, plasmin activation, and the ability of ATRA to inhibit serous ovarian cancer cell survival and invasion. We also employed for the first time an ex vivo tissue explant assay to assess response to ATRA treatment in serous ovarian cancers.

## Methods

### Cell culture

Serous ovarian cancer cell lines COV362, COV318 and OAW28, were purchased from the European Collection of Cell culture (ECCC, Cell Bank Australia) in November 2014. OVCAR-3 were purchased from American Type Culture Collection (ATCC, VA, USA) in October 2016. OV-90 cells were obtained from ATCC in 2008 and authenticated by short tandem repeat (STR) DNA profile on 05/05/2016. COV362, COV318 and OAW28 cell lines were grown in DMEM medium (Life Technologies, Carlsbad, CA, USA) and supplemented with 10% foetal calf serum (FCS) (Sigma-Aldrich St Louis, MO, USA). OV-90 cell line was grown in RPMI 1640 medium supplemented with 10% FCS whilst OVCAR-3 cell line was supplemented with 5% FCS. All cell lines were cultured with antibiotics (100 U penicillin G, 100 μg/ml streptomycin sulphate and 0.25 μg/ml amphotericin B, Sigma Aldrich) and maintained at 37 °C in 5% CO_2_ environment and routinely checked for mycoplasma contamination.

Primary ovarian cancer cells were derived from ascites collected from advanced stage serous ovarian cancer patients as described previously [32]. All primary cells were grown in advanced RPMI 1640 medium (cat no 12633–020, Life Technologies) supplemented with 4 mM L-glutamine, 10% FCS (Sigma Aldrich) and antibiotics (100 U penicillin G, 100 μg/ml streptomycin sulfate and 100 μg/ml amphotericin B, Sigma Aldrich). The clinicopathological characteristics of the patients whose ascites was used to isolate the primary cells are shown in Additional file 1: Table S1.

### Cell survival assay

Ovarian cancer cell lines (OVCAR-3, OAW28, COV318, COV-362, OV-90) and primary serous ovarian cancer cells (*n* = 6 patients) were plated at 5000 cells/well in a 96 well plate and treated in quadruplicates with ATRA (1 & 5 μM) (R2625, Sigma-Aldrich) or control medium containing 0.1% DMSO. The medium was changed after 3 days with media containing either ATRA or 0.1% DMSO (solvent control). After 6 days treatment, conditioned media (CM) was removed and 3–(4,5-dimethylthiazol-2-yl)-2,5-diphenyltetrazolium bromide (MTT) (5 mg/mL 1:10 at respective medium, Sigma-Aldrich) was added and cells were incubated for 4.5 h at 37 °C. Following the addition of MTT solvent (0.1 N HCl in isopropanol), absorbance readings were measured at 595 nm using the Triad series multimode detector plate reader (Dynex technologies, VA, USA) as described previously [33].

### Plasmin activation assay

Serous ovarian cancer cells (OAW28, OV-90 & OVCAR-3) were plated in 96 well plates (5000 cells/well) and treated with ATRA (1-5 μM) for 6 days. Cells were washed with 0.1 M phosphate buffered saline (PBS), pH 7.4 and treated for 10 min ± 0.5 μM plasminogen (P7999, Sigma Aldrich) and in the presence or absence of plasmin inhibitor (ε-aminocaproic acid; ε-ACA, 100 mM, Sigma Aldrich) prior to the addition of the plasmin substrate, chromozyme PL (3 mM in 100 mM glycine, Roche Diagnostics, Mannhein, Germany). Plasmin activity was measured at 405 nm using the Triad series multimode detector (Dynex Technologies, VA, USA) over a 2 h period as described previously [6]. The colorimetric change resulting in the formation of yellow *p*-nitroaniline is a direct measure of the plasmin activity. Plasmin (0.1 U/ml, P1867, Sigma Aldrich) was used as a positive control and negative controls included parallel wells containing no chromozyme PL substrate and growth media alone, which served as the reagent blank.

### Cell motility and invasion

Ovarian cancer cell lines (OAW28 & OV-90) were treated with ATRA (1 and 5 μM) for 6 days in 75cm^2^ flasks. Cells were trypsinized, labelled with calcein-AM (1 μg/ml, Invitrogen) and added on to uncoated 12 μm filters inserts (96 well plate, Chemo Tx, Neuro Probe, MD, USA) for motility assays or 12 μm filters coated with Geltrex (0.6 μl/well, Life Technologies) for invasion assays. FCS (10%) was used as chemoattractant in the bottom chamber for both the motility and invasion assays. After 6 h, cells that migrated or invaded into the lower chamber were measured. The fluorescence was measured at 485–520 nm using the Triad series multimode detector (Dynex Technologies, VA, USA) as previously described [8].

### RNA extraction and quantitative real-time PCR

Ovarian cancer cell lines (OAW28, OV-90) were plated at 10–15,000 cells/well in a 96 well plate and treated in triplicate with ATRA (0- 5 μM) for 1–6 days. Total RNA was isolated and reverse transcribed using the TaqMan® Gene expression Cells-to-CT™ kit (Applied Biosystems, Mulgrave, Victoria, Australia), as per the manufacturer’s instructions as described previously [32]. Resultant cDNA was stored as 50 μl aliquots at − 20 °C for qRT-PCR analysis. qRT-PCR reactions were performed on cDNA samples using TaqMan® primer sets for *ANXA2* (Hs00743063_s1) and *S100A10* (Hs00751478_s1) using the Quantstudio 12 K Flex Real Time PCR System (Applied Biosystems). PCR cycling conditions were as follows: 50 °C for 2 min, 95 °C for 10 min followed by 40 cycles of 95 °C for 15 s and 60 °C for 1 min. CT values were normalised to the house keeping gene β-actin (4333762F, Applied Biosystems) using the 2^-∆∆CT^ method. β-actin CT values were not altered by ATRA treatment (data not shown).

### Western blotting

Ovarian cancer cell lines (OAW28, OV-90) were treated with ATRA (1, 5 μM) for 6 days to 80% confluence in 75cm^2^ flasks. Cells were dislodged using a cell scraper and cell pellet were resuspended in 200 μl of RIPA buffer (1% Nonidet P-40, 1% sodium deoxycholate, 0.1% SDS, 0.15 M sodium chloride, 50 mM Tris- HCL and 1 mM EDTA, pH 8.0 with protease inhibitor) spun at 7000 rpm for 10 min and stored at − 20 °C. Equal amounts of protein were electrophoresed and transferred to PVDF membranes (GE Healthcare, *Little Chalfont,* Buckinghamshire, UK) as described previously [6]. Proteins bands were detected with mouse monoclonal antibodies to annexin A2 (1/2000, Clone 5, 610069, BD Biosciences, North Ryde, NSW, Australia) or S100A10 (1/2000, Clone 148, 610070, BD Biosciences), anti-mouse IgG peroxidase-conjugated secondary antibody (1/4000, A0168, Sigma Aldrich), enhanced chemiluminescence (GE Healthcare), and ChemiDoc™ MP Imaging System with ImageLab™ software (Bio-Rad, Hercules, CA, USA) [8]. β-actin, used as a loading control was detected using a rabbit polyclonal antibody to β-actin (1/2000, ab8227, Abcam, Cambridge, MA, USA) and anti-rabbit IgG peroxidase-conjugated secondary antibody (1/4000, AP132P, Merck, Millipore, Bayswater, VIC, Australia).

### Immunocytochemistry

Ovarian cancer cells (OAW28 & OV-90) were plated (10,000–15,000 cells/well) in 8 well tissue culture chamber slides (Nunclon™ Lab-Tek II Chamber slide, RS Glass Slide, Naperville, IL) in 500 μl 10% FCS RPMI for 24 h and treated with control medium (0.1% DMSO) or ATRA (5 μM). The medium was changed after 3 days treatment with either control medium or medium containing ATRA (5 μM). After 6 days treatment, cells were washed with cold PBS (3x) and fixed with cold 100% methanol (3 min) and cold 100% acetone (1 min), washed with PBS (2 × 5 min), blocked with 5% goat serum and incubated overnight with mouse monoclonal annexin A2 (1/100, BD Biosciences) or S100A10 (1/200, BD Biosciences) antibodies. Annexin A2 or S100A10 was visualized with goat anti-mouse Alexa Fluor ® 488 or goat anti-mouse Alexa Fluor ® 594 for 1 h at RT, (1/200, Molecular Probes, Life Technologies) respectively, and slides were mounted with ProLong Gold Antifade Mountant with DAPI (P36931, Molecular Probes, Life Technologies). Cells were viewed with an epifluorescence microscope (BX50, Olympus, Tokyo, Japan) and imaged using a 40x objective and a Spot RT digital camera (Diagnostic Instruments, Sterling Heights, MI). Negative controls included mouse immunoglobulin or no primary antibody. The percentage of cells with membrane staining in control and ATRA treated cells were determined visually by an assessor that was blinded to the treatment groups. To calculate the % of positive cell with membrane staining, cells (~ 200–300) in five high power images were scored visually for the presence or absence of annexin A2 or S100A10 membrane staining.

### Ex vivo tissue explant assay

Cryopreserved serous ovarian tissues stored in liquid nitrogen (*n* = 12) were thawed, dissected into 1-mm^3^ pieces, and explanted onto gelatine dental sponges (Spongostan, Johnson & Johnson, Humanus Dental AB, Malmo, Sweden) immersed in RPMI-1640 media supplemented with 10% FCS and antibiotics as described previously [34]. Tissue explants were incubated with control media (0.1% DMSO) or ATRA (1 μM) in a humidified atmosphere at 37 °C containing 5% CO_2_, collected and fixed with 10% formalin (Sigma-Aldrich) after 72 h treatment. The clinical and pathological characteristics of the patients used for explant culture are summarized in Additional file 2: Table S2.

### Immunohistochemistry

Effects of ATRA on proliferation and apoptosis in explant tissues were assessed by immunohistochemistry as described previously [34]. Tissue sections were incubated over night at 4 °C with rabbit monoclonal antibody to Ki67 (1/400, M3060, Taylor Biomedical Pty Ltd., NSW, Australia) or rabbit polyclonal antibody to cleaved caspase 3 (1/200, 9661 L, Cell Signalling). The following day, the slides were incubated with biotinylated goat anti-rabbit (1/400, E0432018 Dako, North Sydney, NSW, Australia) and streptavidin-HRP (1/500, P0397 Dako) for 1 h at room temperature. Diaminobenzidine/H_2_O_2_ (Sigma Aldrich), substrate was used to detect immunoreactivity before counterstaining with 10% haematoxylin (Sigma Aldrich), dehydrating and mounting in Pertex (Medite Medizintechnik, Germany). Additional sections were immunostained with annexin A2 and S100A10 antibodies as described previously [9]. Slides were digitally scanned using the NanoZoomer (Hamamatsu Phontonics K.K, Hamamatsu, Japan). Up to 5–8 cancer tissue areas were randomly selected and cells positive for Ki67 and cleaved caspase 3 were counted by two independent assessors who were blinded to the treatment [34]. Total annexin A2 and S100A10 including membrane and cytoplasmic staining was measured by video image analysis (VideoPro 32; Leading Edge P/L, Marion, South Australia, Australia) as described previously [35]. Measurements of the diaminobenzidine (DAB) stained area (i.e. positively stained area in pixel units) and the total tumor area examined (i.e. positively and negatively stained area in pixel units) from 5 to 10 high power fields were used to derive the % positive area (% POS).

### Statistical analysis

All statistical analyses were performed using GraphPad Prism (version 7.02, CA, USA). The Student’s t-test or one-way ANOVA was used to assess statistical significance between control and treatment groups (RT-PCR, MTT assay, motility and invasion assays). The Friedman pair test was used to assess differences between control and ATRA treatment groups in the MTT assay using the primary serous ovarian cancer cells. The Wilcoxon rank paired test was used to assess differences between control and ATRA treatment groups in the ex vivo explants assays*.* The null hypothesis is that ATRA treatment has no effect. Statistical significance was accepted at *p* < 0.05.

## Results

### Effects of ATRA treatment on serous ovarian cancer cell survival

**Fig. 1:**
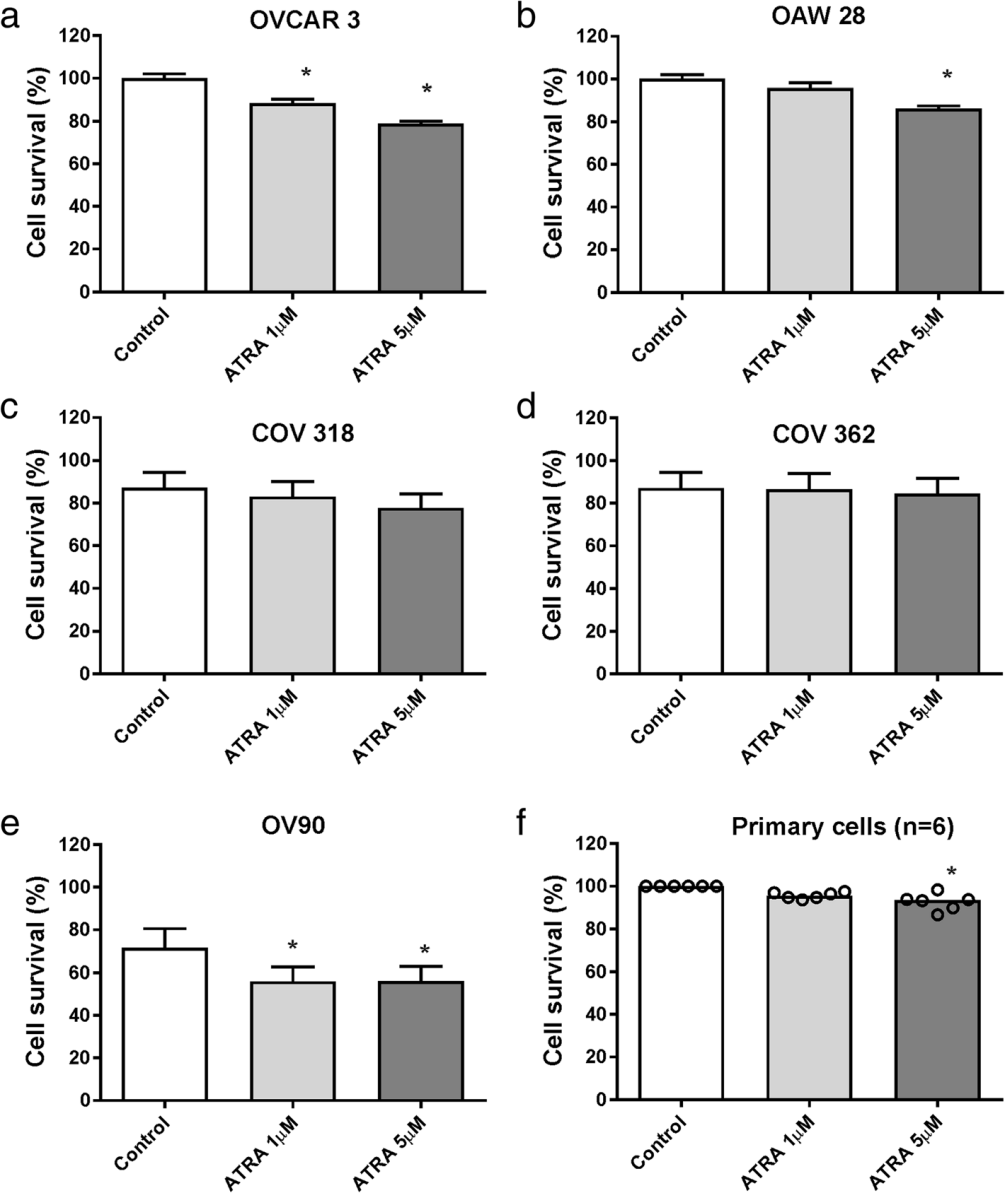
The effect of ATRA treatment on serous ovarian cancer cell proliferation. Cell survival in response to 6 days ATRA treatment (1, 5 μM) is shown in order of cell invasiveness **a** OVCAR-3, **b** OAW28, **c** COV-318, **d** COV- 362, **e** OV-90 and primary serous ovarian cancer cells (*n* = 6) (**f**). Data are expressed as percentage of control, mean ± SEM from 3 to 4 independent experiments (20–24 determinations) in (**a**-**e**) and quadruplicate determinations for primary cells from 6 individual patients in (**f**). Statistical significance for a-e was determined using one-way ANOVA (Tukey multiple comparison post hoc test) and the paired Friedman test (Dunn’s multiple comparison test) for (**f**), *p < 0.05

Survival of OVCAR-3 (Fig. 1a), OAW28 (Fig. 1b) and OV-90 (Fig. 1e) cells was significantly reduced after 6 days of ATRA (5 μM) treatment but no significant effects were observed on COV318 (Fig. 1c) or COV362 cells (Fig. 1d). The most sensitive cell lines were OV-90 (cell survival reduced to 78.6% of control with 5 μM ATRA) and OVCAR-3 (78.8% of control) followed by OAW28 (87.6% of control). ATRA treatment (5 μM) for 6 days also had a significant effect on the survival of the primary serous ovarian cancer cells (*n* = 6) isolated from patient’s ascites (range 86.7–98% of control, Fig. 1f, *p* = 0.0017). Response to ATRA treatment in the ovarian cancer cell lines did not correlate with expression of *ANXA2, S100A10* or retinoic acid receptor alpha *(RARA)* as all cell lines exhibited similar gene expression levels (Additional file 3: Figure S1a). Similarly there was no relationship between ATRA response and ratio of two genes *CRABP2/FABP5*, intracellular lipid binding proteins, previously shown to be associated with retinoic acid induced growth inhibition [36] (Additional file 3: Figure S1b). Three of the serous ovarian cancer cell lines (OVCAR-3, OAW28 and OV-90) that were growth-inhibited by ATRA treatment were selected and investigated in further studies below.

### Effect of ATRA treatment on plasmin generation, ovarian cancer cell motility and invasion

**Fig. 2:**
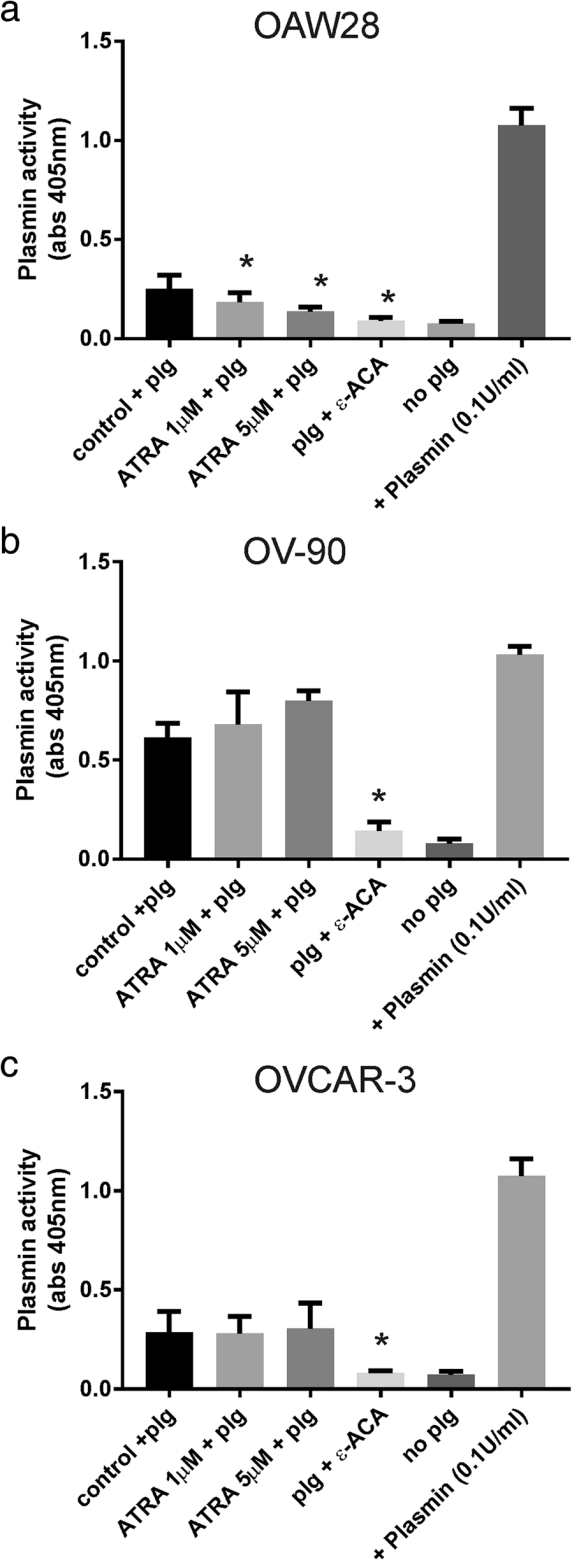
The effect of ATRA treatment on plasmin generation. Effect of 6 days ATRA treatment (5 μM) on plasmin activation in OAW28 (**a**), OV-90 (**b**), and OVCAR-3 (**c**) cells. Plg = plasminogen, ε-ACA = ε-aminocaproic acid. Data are the absorbance readings at 405 nm from 2 to 3 independent experiments (4–10 determinations). Statistical significance was determined using one-way ANOVA and the Tukey multiple comparison post hoc test, *p < 0.05

**Fig. 3:**
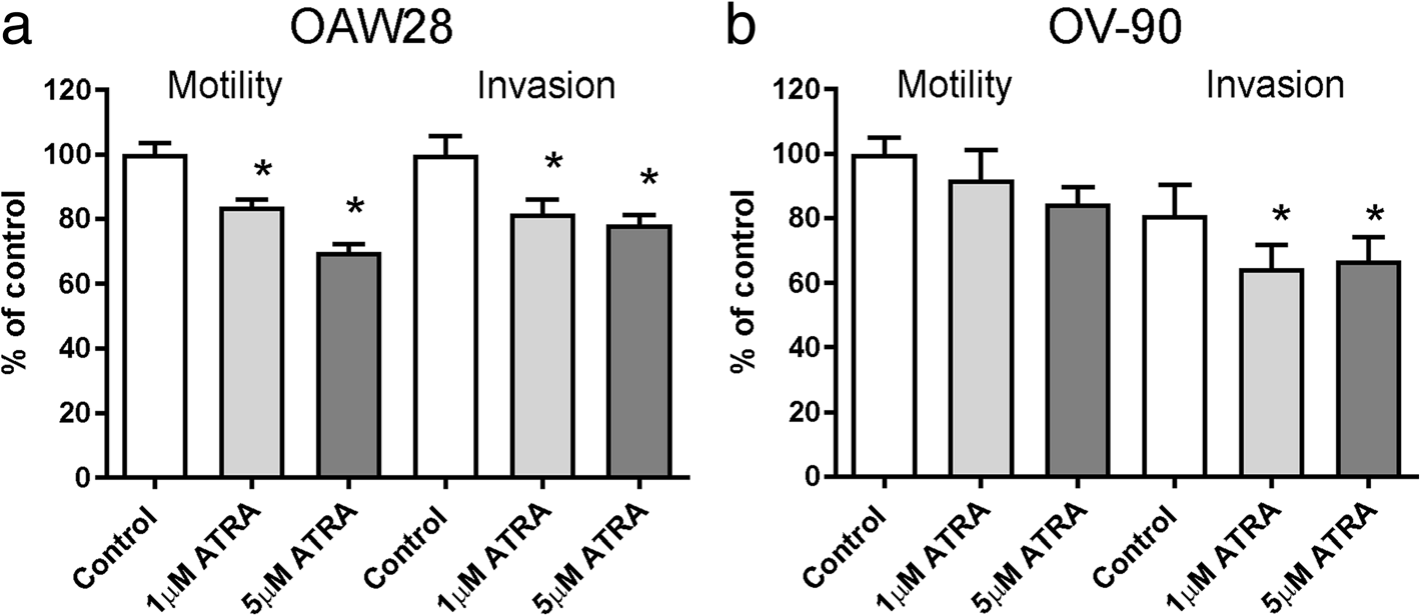
Effect of ATRA treatment on motility and invasion. Effect of 6 days ATRA treatment (1, 5 μM) on motility and invasion of OAW28 (**a**) and OV-90 cells (**b**). Data are expressed as percentage of DMSO control, mean ± SEM from 3 to 5 independent experiments (10–20 determinations). Statistical significance was determined using one-way ANOVA and the Tukey multiple comparison post hoc test, *p < 0.05

ATRA (1 and 5 μM) treatment significantly inhibited plasmin generation in OAW28 (~ 45% of control, Fig. 2a) but did not significantly affect plasmin activation in OV-90 cells (Fig. 2b). ATRA treatment also had no significant effect on plasmin activation in OVCAR-3 cells (Fig. 2c). However in the presence of plasmin inhibitor, ε-ACA, plasmin generation was significantly inhibited in OAW28, OV-90 and OVCAR-3 cells (Fig. 2a-c). We next examined the effects of ATRA on motility and invasion of OAW28 and OV-90. Both OAW28 motility (Fig. 3a, *p* < 0.0001) and invasion (Fig. 3a, *p* = 0.0008) were inhibited by 6 days of ATRA (1 and 5 μM) treatment. Invasion but not motility of OV-90 cells was significantly inhibited by 6 days of ATRA (1 and 5 μM) treatment (Fig. 3b, *p* = 0.0073).

### Effects of ATRA on annexin A2 and S100A10 expression in serous ovarian cancer cell lines

**Fig. 4:**
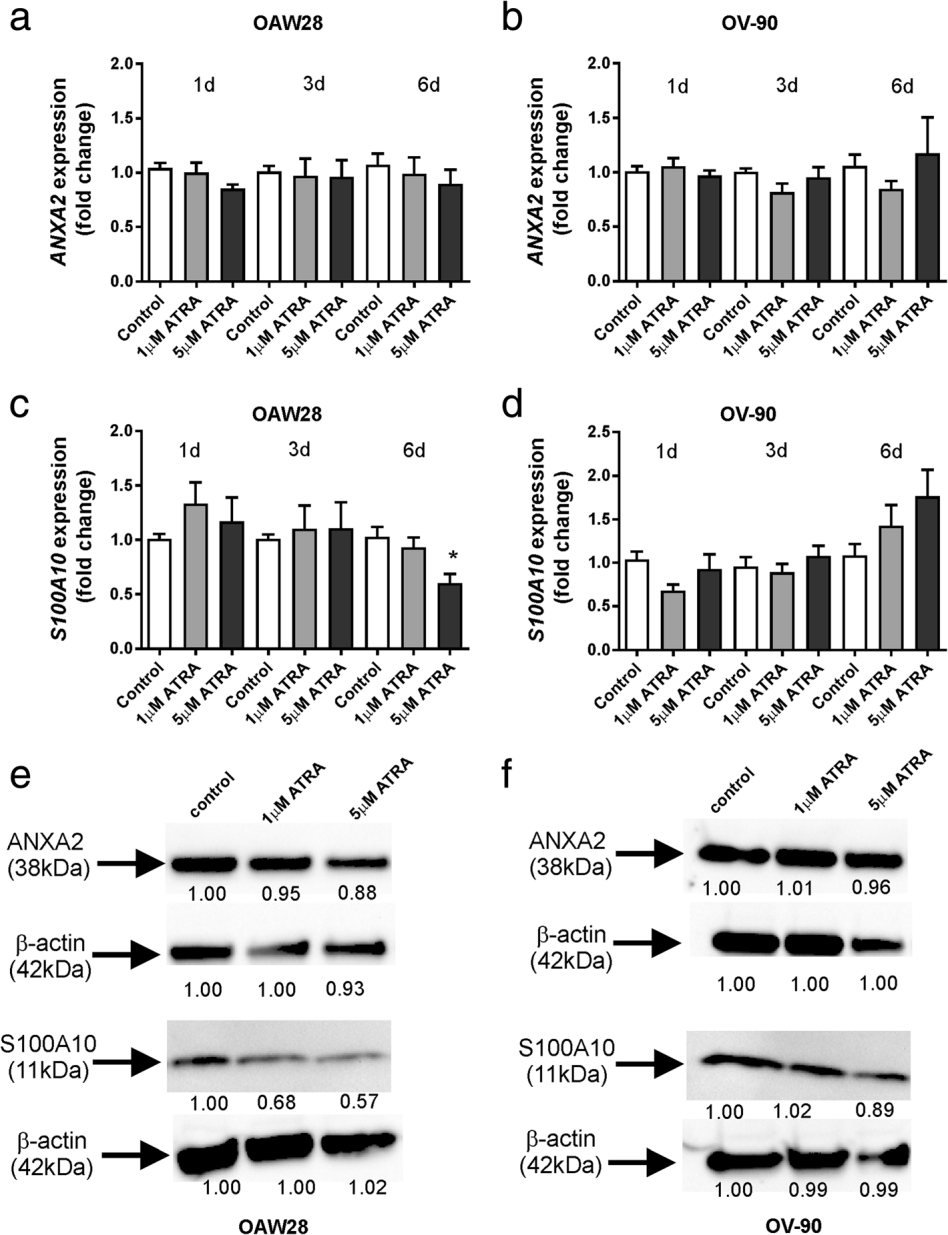
The effect of ATRA treatment on expression of ANXA2 and S100A10 mRNA and protein levels. The effect on *ANXA2* and *S100A10* expression following 1–6 days ATRA treatment (1, 5 μM) in OAW28 (**a** and **c**) and OV-90 (**b** and **d**) cells. *ANXA2* and *S100A10* expression was assessed using 2^-∆∆CT^ quantitation method and normalised to housekeeping gene β-actin using no treatment control as a calibrator. Data is from 2 to 4 independent experiments (*n* = 4–12). Statistical significance was determined using one-way ANOVA and the Tukey multiple comparison post hoc test. Western blots for annexin A2 and S100A10 (**e** and **f**) following 6 days ATRA treatment (1, 5 μM). Annexin A2 bands at 38 kDa, S100A10 at 11 kDa and β-actin bands at 42 kDa are shown for OAW28 (**e**) and OV-90 (**f**) cells. Equal amounts protein (5 μg for annexin A2 & 15 μg S100A10 were run on a 4–12% SDS-PAGE gel and immunoblotted with mouse monoclonal antibodies to annexin A2 (1/2000, BD Biosciences) or S100A10 (1/2000, BD Biosciences). A rabbit polyclonal antibody was used detect β-actin (1/2000, ab8227, Abcam). Numbers below western bands in e and f are fold changes relative to the control treatment. Westerns blots are representative of two independent experiments

**Fig. 5:**
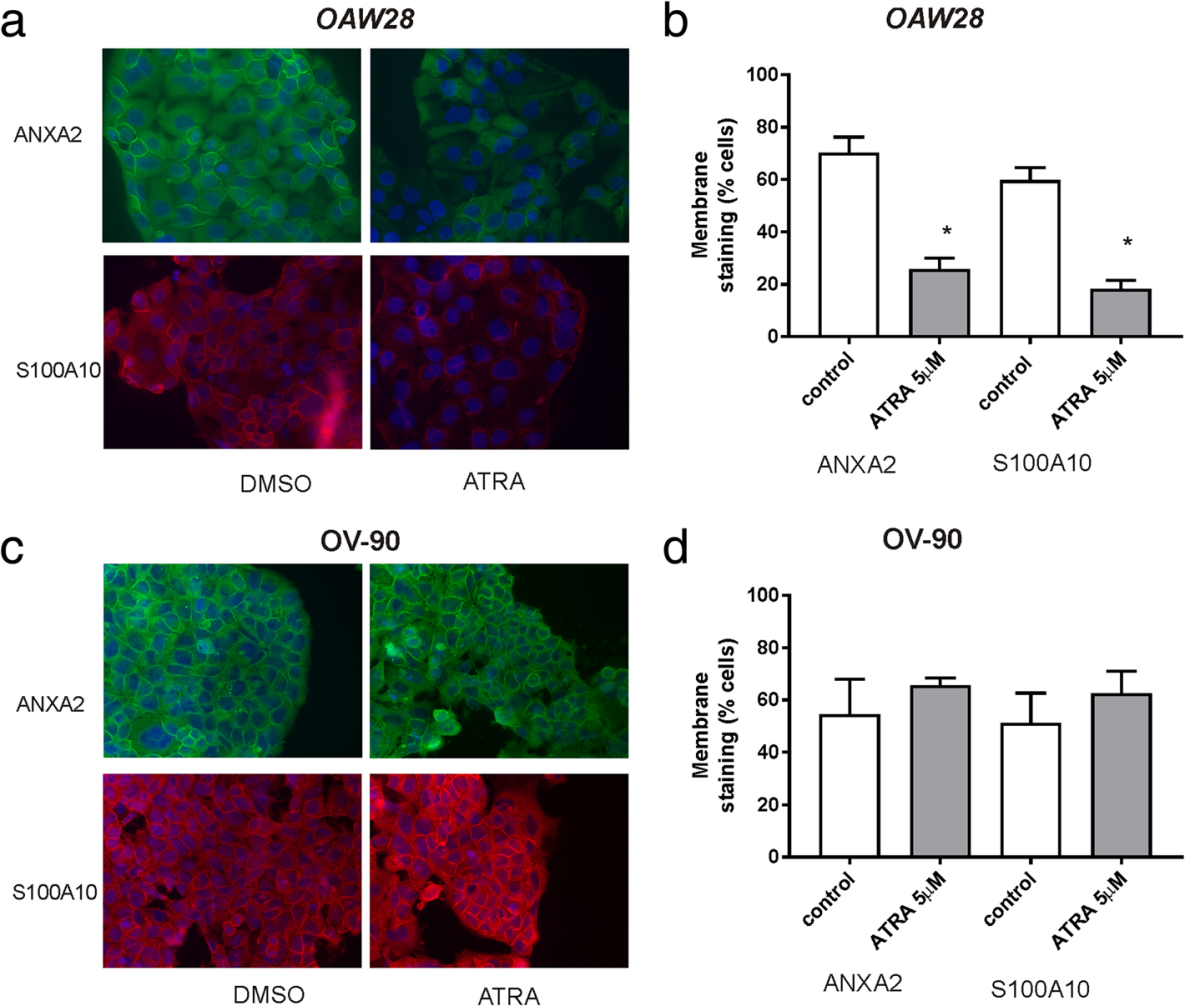
Effect of ATRA treatment on membrane localization of ANXA2 and S100A10 in OAW28 and OV-90 cells. ANXA2 and S100A10 immunofluorescence in OAW28 (**a**) and OV-90 (**c**) cells ± treatment with ATRA (5 μM). ANXA2 and S100A10 membrane localization is significantly reduced in OAW28 cells (**b**) but not OV-90 cells (**d**) following 6 days ATRA treatment. Data is expressed as mean % membrane positive cells ± SEM from 3 independent experiments. * *p* < 0.05 (unpaired student t test)

We examined the effects on ATRA treatment on the expression of both *ANXA2* and *S100A10* in OAW28 and OV-90 cells by qRT-PCR. No significant effect on *ANXA2* expression was observed in OAW28 or OV-90 cell lines (Fig. 4a and b) following 1–6 days of ATRA (1 μM or 5 μM) treatment. However, ATRA treatment (5 μM) for 6 days significantly reduced the expression of *S100A10* in OAW28 (Fig. 4c) but not OV-90 cells (Fig. 4d). We performed western blotting to determine if ATRA had effects on annexin A2 and S100A10 protein levels. An annexin A2 band at 38 kDa and an 11 kDa band for S100A10 was observed in both cell lines, respectively (Fig. 4e and f). ATRA treatment reduced annexin A2 protein levels in OAW28 (88% of control with 5 μM ATRA, Fig. 4e) but had a minimal effect on annexin A2 protein levels in OV-90 cells (96% of control with 5 μM ATRA, Fig. 4f). ATRA treatment markedly reduced S100A10 protein levels in OAW28 (57% of control with 5 μM ATRA, Fig. 4e) but had minimal effect on S100A10 protein levels in OV-90 cells (89% of control with 5 μM ATRA, Fig. 4f). ATRA treatment (5 μM) significantly reduced the proportion of OAW28 cells exhibiting membrane annexin A2 (Fig. 5a, *p* = 0.004) and S100A10 (Fig. 5b, *p* = 0.002) but did not significantly affect membrane localization of annexin A2 or S100A10 in OV-90 cells (Fig. 5c and d).

### Effect of ATRA treatment using ex vivo serous ovarian cancer tissue explant assay

**Fig. 6:**
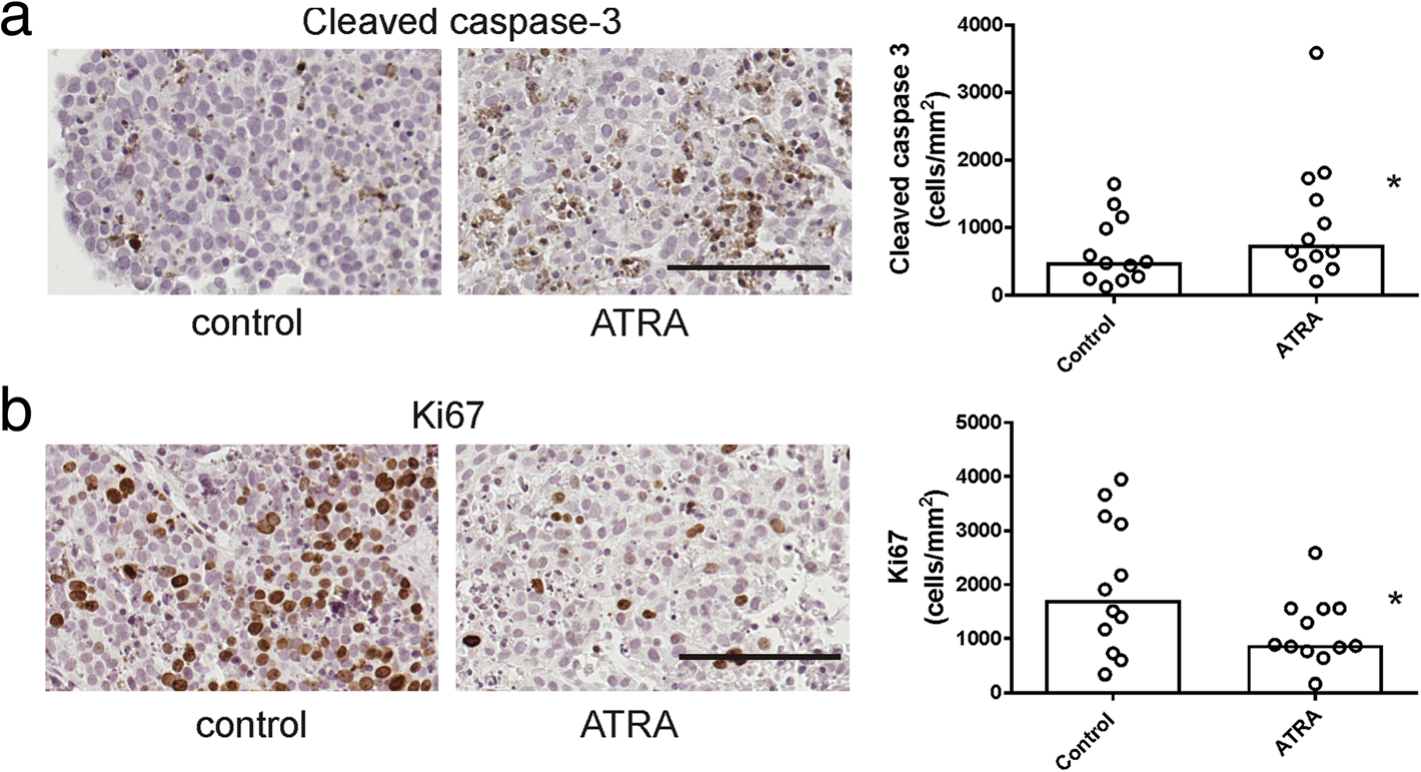
The effect of ATRA treatment on proliferation and apoptosis in ex vivo tissue explants. High grade serous ovarian cancer tissues (*n* = 12) were treated with control medium (0.1% DMSO) or ATRA (1 μM) for 72 h. Apoptosis was assessed using an antibody to cleaved caspase 3 (**a**) and proliferation was assessed using Ki67 antibody (**b**). Representative images for cleaved caspase 3 and Ki67 are shown (scale bar = 100 μm, all images are the same magnification). The summary of the immunostaining quantitation for cleaved caspase 3 (**a**) and Ki67 (**b**) is shown. The data represents the number of Ki67 or cleaved caspase 3 positive cells/mm^2^ for each tissue. The bar is the median value. *p < 0.05, Wilcoxon pair test

**Fig. 7:**
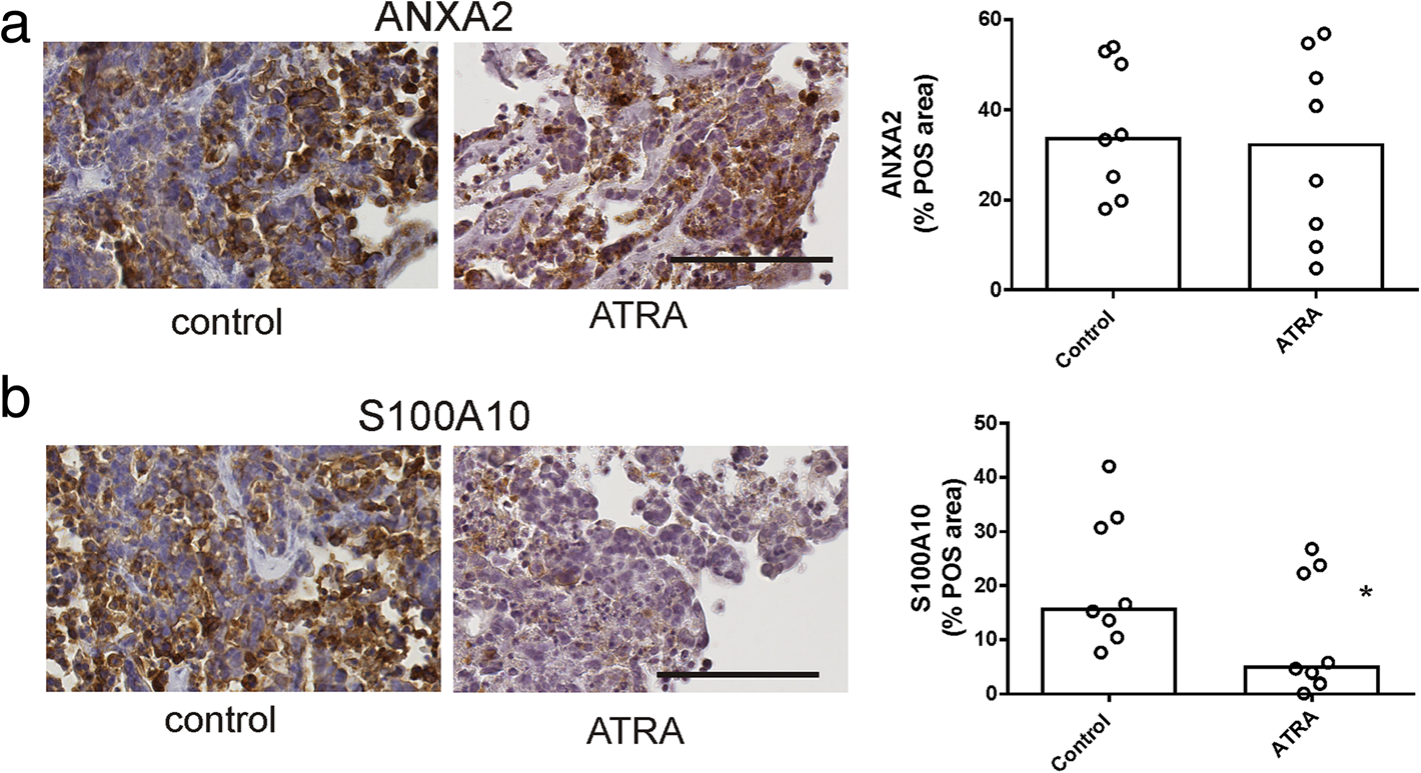
The effect of ATRA treatment on annexin A2 and S100A10 protein levels in ex vivo tissue explants. High grade serous ovarian cancer tissues were treated with control medium (0.1% DMSO) or ATRA (1 μM) for 72 h (*n* = 8). Representative images for annexin A2 (**a**) and S100A10 (**b**) immunostaining are shown (scale bar = 100 μm, all images are the same magnification). The summary of the annexin A2 and S100A10 immunostaining quantitation for 8 patients are shown. The data represents the area of annexin A2 or S100A10 positivity measured by video image analysis (% positive area). The bar is the median value. *p < 0.05, Wilcoxon pair test

The effect of ATRA treatment (1 μM) on ex vivo high grade serous ovarian cancer tissues was assessed by immunohistochemistry using antibodies to cleaved caspase 3 (Fig. 6a) and Ki67 (Fig. 6b), respectively. ATRA treatment significantly increased the number of positive cleaved caspase 3 cells (Fig. 6a, *p* = 0.0024, Wilcoxon rank test) and significantly reduced Ki67 positivity (Fig. 6b, *p* = 0.0034, Wilcoxon rank test) in the serous ovarian cancer explant tissues. Effects of ATRA on apoptosis and proliferation were associated with a significant reduction in total S100A10 protein levels by immunohistochemistry (Fig. 7b, *p* = 0.0273, Wilcoxon rank test). However total annexin A2 protein levels were not altered by ATRA treatment (Fig. 7a, *p* = 0.156, Wilcoxon rank test).

## Discussion

ATRA is used clinically to improve bleeding symptoms in patients with APL [20]. There has been great interest in expanding the therapeutic use of ATRA to other cancers due to its low toxicity [37, 38]. In this study we show that ATRA treatment: *1*) inhibited cell survival in 3 serous ovarian cancer cell lines (OVCAR-3, OAW28 & OV-90), *2*) reduced proliferation of primary serous ovarian cancer cells derived from patient ascites, *3*) inhibited plasmin production by OAW28 but not OV-90 or OVCAR-3 cells, *4*) reduced S100A10 expression and membrane localization of both S100A10 and annexin A2 in OAW28 cells but not OV-90 cells, *5*) inhibited invasion of both OAW28 and OV-90 cells, *6*) increased apoptosis and decreases proliferation in ex vivo high grade serous ovarian cancer tissues and *7)* reduced S100A10 but not annexin A2 protein levels in ex vivo high grade serous ovarian cancer tissues. Together our findings indicate that ATRA has promising potential as a novel therapeutic against high grade serous ovarian cancer.

ATRA was effective at inhibiting cell survival by more than 20% in 2 of 5 serous ovarian cancer cell lines (OVCAR-3, OV-90) and was very effective at increasing apoptosis and decreasing proliferation in serous ovarian cancer tissues in the ex vivo tissue explant assay. These findings are supported by previous in vitro ovarian cancer studies that have shown ATRA treatment inhibited growth or cell cycle progression of a range of ovarian cancer cell lines including ES2, A2780, CP70, OVCAR-3, MDAH-2774, CAOV3 and HOC-7 [27–31, 39]. Wu et al. (1997) and Soprano et al. (2006) showed that ATRA (10^− 6^ to 10^− 10^ M) inhibited cell cycle progression at the G1 stage of the cell cycle in CAOV-3 cells but had no effect on a highly invasive ovarian cancer cell line, SKOV-3 following 7 to 15 days of ATRA treatment [28, 31]. Krupitza et al. (1995) showed effects of ATRA (40 to 140 nm) alone induced HOC-7 cell apoptosis and decreased cell viability [29]. Similarly, Karabulut et al. (2010) showed ATRA (10 μM) suppressed cell growth and increased cell apoptosis in two ovarian cancer cell lines (OVCAR-3 and MDAH-2774) [27].

A recent study using network guided modelling in more than 30 tumor types identified an ATRA-21 gene signature that has potential to be used to predict response to ATRA in individual patients [26]. The ATRA-21 gene signature correctly identified APL as the most sensitive leukaemia subtype to respond to ATRA treatment but also identified patients with high sensitivity prediction scores with many other tumor types that may also benefit from ATRA treatment [26]. Interestingly, up to 29% (117/402) of serous carcinoma patients exhibited a high sensitivity prediction score (> 0.266) similar to AML patients [26]. The sensitivity prediction score using the ATRA-21 signature significantly correlated with ATRA response in the Genomic Drug Sensitivity Cancer (GDSC) project. Two of 13 ovarian cancer cell lines (OAW28 and A2780) reached the 20% reduction in cell number threshold in the GDSC study [26]. Other serous ovarian cancer cell lines investigated in this study including OVCAR-3, COV318, COV362 and OV-90 were not included in both the ATRA 21-prediction modelling and the GDSC project [26].

We found that serous ovarian cancer cells COV362 and COV318 were resistant to ATRA treatment. In previous studies, SKOV-3, and CP70 ovarian cancer cells have also been shown to be resistant to ATRA [28, 39]. Although is not clear why ovarian cancer cell lines are resistant to ATRA treatment, our study suggests that resistance to ATRA treatment is not dependent on *RARA*, *ANXA2* or *S100A10* expression as all of the serous ovarian cancer cell lines expressed similar mRNA levels of these genes (Additional file 3: Figure S1a). Moreover, ATRA treatment response is not associated with the invasive potential of serous ovarian cancer cells as the survival of both less invasive (OVCAR-3, OAW28) and the more highly invasive serous ovarian cancer cell line (OV-90) was inhibited by ATRA treatment. A recent study reported that ovarian cancer cell lines with a high ratio of intracellular lipid binding proteins *CRABP2/FABP5*, involved in retinoic acid delivery from the cytoplasm to nucleus [36] were growth inhibited by 7 days ATRA (7 μM) treatment [40]. Our results demonstrating anti-proliferative effects of ATRA in OAW28 cells which exhibited the highest *CRABP2/FABP5* ratio agree with these findings however, this notion is not supported in OV-90 cells which exhibited the lowest *CRABP2/FABP5* ratio. The response to ATRA treatment in OV-90 cells may be dependent on the expression of other signaling pathways. Wu et al. (1997) suggested that ATRA blocks the cell cycle progression in G1 stage in CAOV3 cells and targets a downstream process or the event occurring at a point after the insulin/IGF-1, oestrogen and serum signal transduction pathways converge [28]. This is supported by Ravikumar et al. (2007) that showed that ATRA (1 μM) treatment blocks the IGFR-1 signalling pathway in CAOV3 by inhibiting the expression of insulin receptor substrate-1 (IRS-1) [39].

The resistance to ATRA may also be as a result of poor delivery of ATRA into ovarian cancer cells due to its high lipophilic nature or the length of treatment [41, 42]. Interestingly, although there were no effects of ATRA treatment on cell proliferation and apoptosis of SKOV-3 cells in previous studies [28, 39], a recent study by Narvekar et al. (2014) showed ATRA’s pharmaceutical properties could be enhanced by polymer-oil nanostructured carriers in SKOV-3 cells [41]. Oil-soluble ATRA treatment molecules easily permeate across the cell membrane, induced apoptotic cell death and a long-term anti-tumorigenic effect in SKOV-3 cells [41]. Oil soluble formations of ATRA may improve effectiveness of the delivery of ATRA in ovarian cancer cells and should be further evaluated. More recently Young et al. (2015) showed 3 days treatment with ATRA (10 μM) did not affect proliferation of ovarian cancer cell lines (A2780 and CP70) however, a longer ATRA treatment for 28 days was effective at inhibiting A2780 tumour growth in vivo using mouse xenografts [42]. ATRA treatment reduced expression of the stem cell marker, ALDH1 however the study did not examine effects of ATRA on annexin A2 or S100A10 expression [42]. Future studies should also consider longer treatment times to evaluate the effectiveness of ATRA treatment.

ATRA treatment for 6 days treatment significantly reduced the expression of *S100A10* but not *ANXA2* in OAW28. Similarly, S100A10 but not annexin A2 protein levels were reduced by ATRA treatment in OAW28 cells. We also found that ATRA treatment significantly reduced S100A10 but not ANXA2 protein levels in the ex vivo explant assay. It is likely that reduced S100A10 protein levels inhibit the assembly of annexin A2 heterotetramer (AIIt) in the cell membrane. This view is supported by our findings that both S100A10 and annexin A2 are reduced in cell membranes of OAW28 cells following ATRA treatment. The study by O’Connell et al. (2011) also that showed ATRA treatment (1 μM) reduced S100A10 cell surface expression to a greater extent than annexin A2 in leukemia cells [21]. Gladwin et al. (2000) showed that ATRA (10^− 5^ to 10^− 9^ M) treatment for 6–8 days reduced S100A10 protein levels but not *S100A10* mRNA levels in bronchial epithelial cells (BEAS-2B), suggesting the involvement of a post-translational mechanism [43]. A recent study investigated the mechanism whereby ATRA regulates cell surface expression of S100A10 in APL leukemic cells [44]. They found evidence that ATRA can reduce S100A10 protein levels via an ubiquitin-independent manner [44]. Furthermore they also reported that ATRA treatment reduced *S100A10* expression but not *ANXA2* mRNA or protein levels in the MCF-7 breast cancer cells [44]. The study concluded that ATRA can regulate S100A10 levels independently of PML/RARα and annexin A2 [44]. Our findings support these observations and suggest that ATRA acts predominately on S100A10 in the plasminogen activation pathway in serous ovarian cancer cells.

The invasion of both OAW28 and OV-90 cells was inhibited by ATRA treatment. This findings concur with Young et al. (2015) that showed that ATRA (10 μM) treatment for 28 days inhibited cell migration and invasion of two ovarian cancer cell lines (A2780 and CP70) [42]. ATRA treatment has also recently been shown to inhibit the motility and EMT phenotype of SKBR3 breast cancer cells [45]. Interestingly, ATRA inhibited OV-90 cell survival and invasion but did not affect annexin A2 or S100A10 expression or plasmin generation in these cells, whilst ATRA effects on cell survival and motility and invasion were associated with effects on S100A10 expression and plasmin activation in OAW28 cells. Our findings suggest that ATRA acts predominately to reduce S100A10 protein levels and plasmin production in OAW28 cells, however ATRA effects in OV-90 cells appear to be independent of its effects on the plasminogen activation pathway. It is likely that ATRA acts via an alternative pathway in OV-90 cells, and requires further investigation.

## Conclusion

In conclusion, ATRA has anti-tumor activity on serous ovarian cancer cell lines in vitro and significantly decreased proliferation and increased apoptosis in ex vivo high grade serous ovarian cancer tissues. ATRA has promising potential as a novel therapy against ovarian cancer and should be further evaluated.

## Additional files

Additional file 1: Summary of clinicopathological characteristics of ovarian cancer patients used to isolate primary ovarian cancer cells from ascites. (DOCX 14 kb)

Additional file 2: Summary of clinicopathological characteristics of ovarian cancer patients used in the ex vivo explant assay. (DOCX 18 kb)

Additional file 3: Gene expression in serous ovarian cancer cell lines. a) Gene expression data for ovarian cancer cell lines obtained from Cancer Cell Line Encyclopaedia. https://portals.broadinstitute.org/ccle. b) Relationship between ATRA response (% cell survival) and *CRABP2/FABP5* ratio in serous ovarian cancer cell lines. (TIF 96 kb)

## Abbreviations

APL: Acute promyelocytic leukemia
ATRA: All trans retinoic acid
CM: Conditioned media
CT: Cycle threshold
DMSO: Dimethyl sulfoxide
EMT: Epithelial to mesenchymal transition
FCS: Fetal calf serum
GDSC: Genomic Drug sensitivity Cancer
IGFR-1: Insulin growth factor receptor 1
IRS-1: Insulin receptor substrate-1
MMP: Matrix metalloproteinase
MTT: 3–(4,5-dimethylthiazol-2-yl)-2,5-diphenyltetrazolium bromide
PBS: Phosphate buffered saline
PCR: Polymerase chain reaction
Plg: Plasminogen
*RARA*: Retinoic acid receptor alpha gene
STR: Short tandem repeat
tPA: Tissue plasminogen activator
ε-ACA: ε-aminocaproic acid
