# Supplementary material for: Anti-tumour effects of all-trans retinoid acid on serous ovarian cancer
Source: J Exp Clin Cancer Res. 2019 Jan 8;38:10. doi: 10.1186/s13046-018-1017-7 (PMC6325857; doi:10.1186/s13046-018-1017-7)
Supplement: Supplementary file 1 — Summary of clinicopathological characteristics of ovarian cancer patients used to isolate primary ovarian cancer cells from ascites. (DOCX 14 kb) [file 13046_2018_1017_MOESM1_ESM.docx]

Additional file 1: **Table S1** Summary of clinicopathological characteristics of the patients whose ascites was used to isolate the primary cells

| **Patient** | **Age at diagnosis**  **(years)** | **Stage**  **at Diagnosis** | **Tumor grade** | **Diagnosis** | **Chemosensitive** |
| --- | --- | --- | --- | --- | --- |
| 1 | 81 | IV | 3 | Serous carcinoma of peritoneum | No |
| 2 | 80 | IIIC | 3 | Primary peritoneal carcinoma | Yes |
| 3 | 69 | IIIA | 3 | Papillary serous carcinoma of the ovary | No |
| 4 | 55 | IIIC | 3 | Serous papillary carcinoma of the peritoneum | No |
| 5 | 60 | IIA | 3 | Serous carcinoma of the ovary | Yes |
| 6 | 65 | IV | 3 | Serous papillary carcinoma of the peritoneum | No |
