# Supplementary material for: Anti-tumour effects of all-trans retinoid acid on serous ovarian cancer
Source: J Exp Clin Cancer Res. 2019 Jan 8;38:10. doi: 10.1186/s13046-018-1017-7 (PMC6325857; doi:10.1186/s13046-018-1017-7)
Supplement: Supplementary file 2 — Summary of clinicopathological characteristics of ovarian cancer patients used in the ex vivo explant assay. (DOCX 18 kb) [file 13046_2018_1017_MOESM2_ESM.docx]

Additional file 2: **Table S2** Summary of clinical and pathological characteristics ovarian cancer patient used in the explant assay.

| **Patient** | **Age**  **at Diagnosis**  **(years)** | **Stage**  **at Diagnosis** | **Tumor grade** | **Diagnosis** | **Chemosensitive** |
| --- | --- | --- | --- | --- | --- |
| 1* | 55 | IIIC | 3 | Serous carcinoma of the peritoneum | No |
| 2 | 66 | IIIC | 3 | Serous carcinoma of the ovary | Yes |
| 3 | 51 | IIIC | 3 | Serous carcinoma of the peritoneum | No |
| 4 | 52 | IIIC | 3 | Serous carcinoma of the ovary | Yes |
| 5 | 60 | IIA | 3 | Serous carcinoma of the ovary | Yes |
| 6 | 66 | IV | 3 | Serous carcinoma of the ovary | Yes |
| 7 | 59 | IA | 3 | Serous carcinoma of the ovary | No chemotherapy |
| 8 | 47 | IIIC | 3 | Serous carcinoma of the ovary | NA |
| 9 | 47 | IIIC | 3 | Serous carcinoma of the ovary | NA |
| 10 | 63 | IIIC | 3 | Serous carcinoma of the ovary | NA |
| 11 | 81 | IIIC | 3 | Serous papillary carcinoma of the ovary | NA |
| 12 | 70 | IIIC | 3 | Serous carcinoma of the ovary | NA |

NA=information not yet available

*same patient as patient 4 in Supplementary Table 1
