# Supplementary figures and images for: Anti-tumour effects of all-trans retinoid acid on serous ovarian cancer
Source: J Exp Clin Cancer Res. 2019 Jan 8;38:10. doi: 10.1186/s13046-018-1017-7 (PMC6325857; doi:10.1186/s13046-018-1017-7)

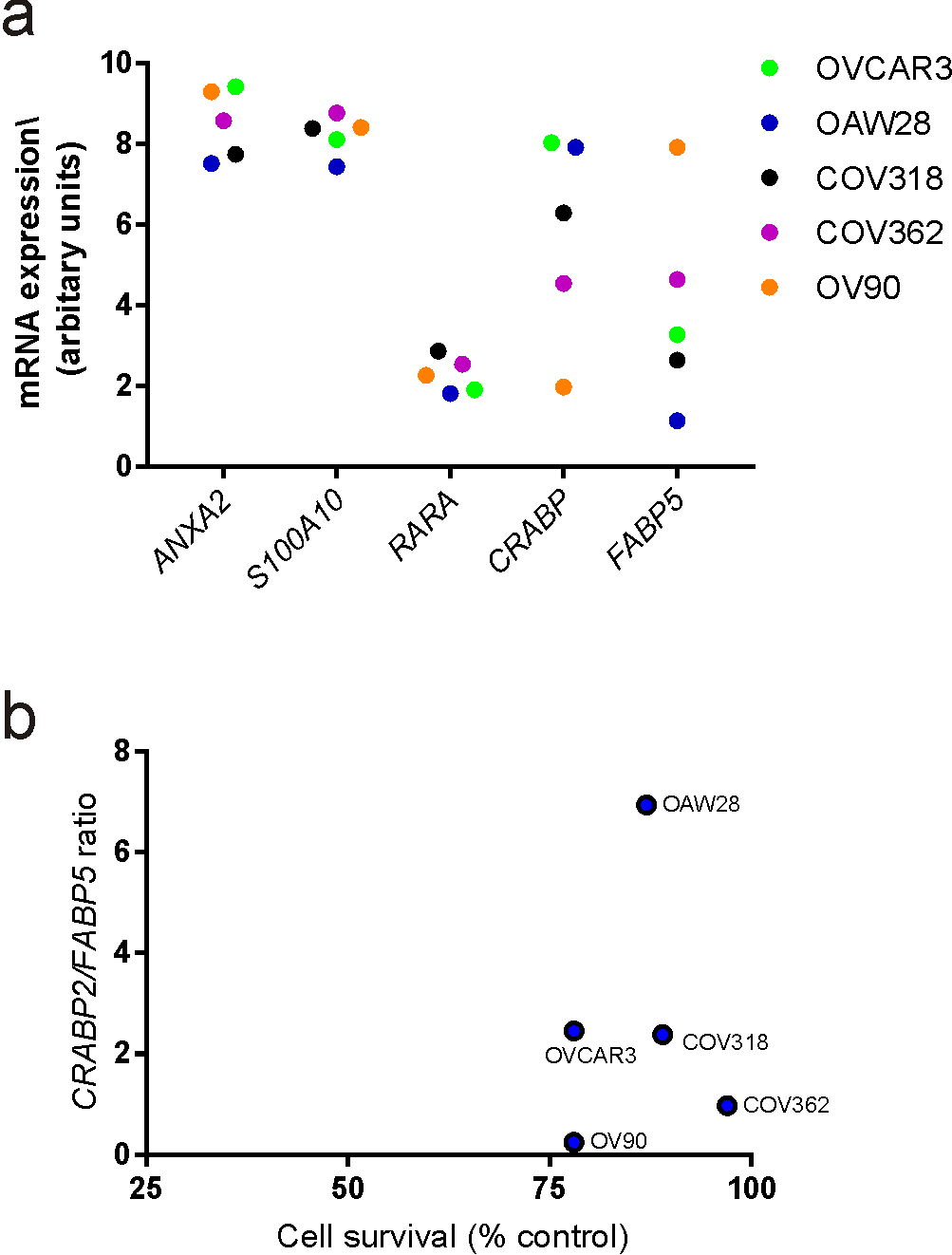

Supplement: Supplementary file 3 — Gene expression in serous ovarian cancer cell lines. a) Gene expression data for ovarian cancer cell lines obtained from Cancer Cell Line Encyclopaedia. https://portals.broadinstitute.org/ccle. b) Relationship between ATRA response (% cell survival) and CRABP2/FABP5 ratio in serous ovarian cancer cell lines. (TIF 96 kb) [file 13046_2018_1017_MOESM3_ESM.tif]
